# Hexokinase 2 upregulation is associated with glycolytic reprogramming and neuroinflammation in hypoxic-ischemic brain damage: a therapeutic target for early intervention

**DOI:** 10.3389/fimmu.2026.1837728

**Published:** 2026-06-12

**Authors:** Yue Li, Chi Qin, Chenxu Miao, Ronghao Mu, Penghua Zhang, Bohao Zhang, Xin Zhao, Xiaoan Zhang

**Affiliations:** 1 Department of Radiology, The Third Affiliated Hospital of Zhengzhou University, Zhengzhou, China; 2 Department of Clinical Research and Translational Medicine, The Third Affiliated Hospital of Zhengzhou University, Zhengzhou, China; 3 Department of Child Developmental Behavior, The Third Affiliated Hospital of Zhengzhou University, Zhengzhou, China

**Keywords:** 3-BrPA, bioinformatics, glycolysis, HIBD, HK2

## Abstract

**Background:**

Hypoxic-ischemic brain damage (HIBD) involves profound metabolic reprogramming, where aberrant glycolysis links to neuronal injury. This study aimed to identify and characterize glycolysis-related hub genes in HIBD.

**Methods:**

Glycolysis-related differentially expressed genes (DEGs) were screened from the HIBD dataset GSE144456 by combining differential expression analysis, weighted gene co-expression network analysis (WGCNA), and a glycolysis gene set. Hub genes were further identified via enrichment and protein-protein interaction (PPI) network analyses and validated in an independent dataset (GSE23317) and our internal RNA-seq. The key hub gene was confirmed in a mouse HIBD model using RT-qPCR and Western blot. To perturb Hexokinase 2 (*Hk2*), 3-bromopyruvate (3-BrPA, 1 mg/kg) was administered 1 hour post-HIBD, and its effects were evaluated by MRI, molecular assays, and behavioral tests.

**Results:**

Bioinformatics analysis identified *Hk2* as a key hub gene that was consistently upregulated in HIBD. *In vivo* experiments demonstrated that both HK2 protein and mRNA levels were significantly elevated 24 hours after HIBD (P < 0.001). Double immunofluorescence staining further revealed that the upregulated HK2 was predominantly localized to Iba1^+^ microglia. Early administration of 3-BrPA reduced acute cerebral infarction volume (P < 0.001), improved neurological function scores (P < 0.001), and concurrently​ downregulated HK2 protein levels (P < 0.05). 3-BrPA treatment also significantly reduced lactate accumulation in the injured brain tissue (P < 0.001). ​It also​ suppressed the mRNA expression of pro-inflammatory cytokines (*Tnf*, *Il1b*, and *Il6*; all P < 0.001) and modulated the protein levels of inflammatory markers (iNOS, ARG1; P < 0.05 and P < 0.01, respectively). Moreover, this single early intervention significantly mitigated long-term brain tissue loss and improved motor coordination and exploratory behavior at 30 days post-injury (P < 0.05).

**Conclusion:**

*Hk2* is highlighted as a critical node associated with both glycolytic reprogramming and neuroinflammation in HIBD, with upregulation primarily in microglia. Early perturbation of glycolysis with 3-BrPA is associated with multifaceted benefits. Our findings link the *Hk2*-glycolysis axis to neuroinflammation, offering a rationale for exploring metabolic interventions in HIBD.

## 1 Introduction

Neonatal hypoxic-ischemic brain damage (HIBD) is a leading cause of neonatal death and long-term neurological disability (1, 2). Although therapeutic hypothermia has improved outcomes to some extent, its narrow treatment time window (typically within 6 hours of birth) and a 40–50% failure rate underscore the urgency of exploring new therapeutic targets (3, 4).

The pathogenesis of HIBD is multifactorial, involving energy failure, excitotoxicity, and neuroinflammation (5). Metabolic reprogramming, particularly a shift towards aberrant glycolysis, is a key pathophysiological event​ in HIBD (6–8). Post-ischemic brain tissue exhibits a classic “aerobic glycolysis” phenotype, leading to lactate accumulation and exacerbated cellular damage (9, 10). Critically, this shift in metabolic state is closely linked to the initiation and amplification of neuroinflammation (11, 12). Studies have shown a coordinated upregulation of key glycolytic enzymes, including hexokinase 2 (*Hk2*), after HIBD, collectively contributing to a pro-inflammatory metabolic microenvironment (13–15). The resulting metabolites (e.g., lactate) not only provide precursors for inflammatory mediator synthesis but can also act as signaling molecules to further amplify inflammatory responses through mechanisms such as post-translational protein lactylation (16, 17).

Given this intimate coupling between metabolism and inflammation, intervening in glycolytic reprogramming during the early phase has emerged as a promising neuroprotective strategy. Preclinical studies have confirmed that glycolytic inhibitors, such as 2-deoxyglucose (2-DG) or 3-bromopyruvate (3-BrPA), can attenuate inflammation and improve outcomes in various brain injury models (18–21). In neonatal HIBD, preliminary evidence also suggests the protective potential of modulating glycolysis-related pathways. Of particular note, recent work in ischemic stroke models has identified *Hk2* in microglia as a key metabolic node driving pro-inflammatory polarization, and its inhibition effectively reduces neuroinflammation (22). This suggests that *Hk2* may serve as a potential hub linking metabolic reprogramming to neuroinflammation (22, 23), rather than merely a passive responder to upstream signaling. Consequently, employing known *Hk2* inhibitors, such as 3-BrPA, to probe the therapeutic potential of targeting this hub and its associated pathway represents a rational approach (24).

**Figure 1:**
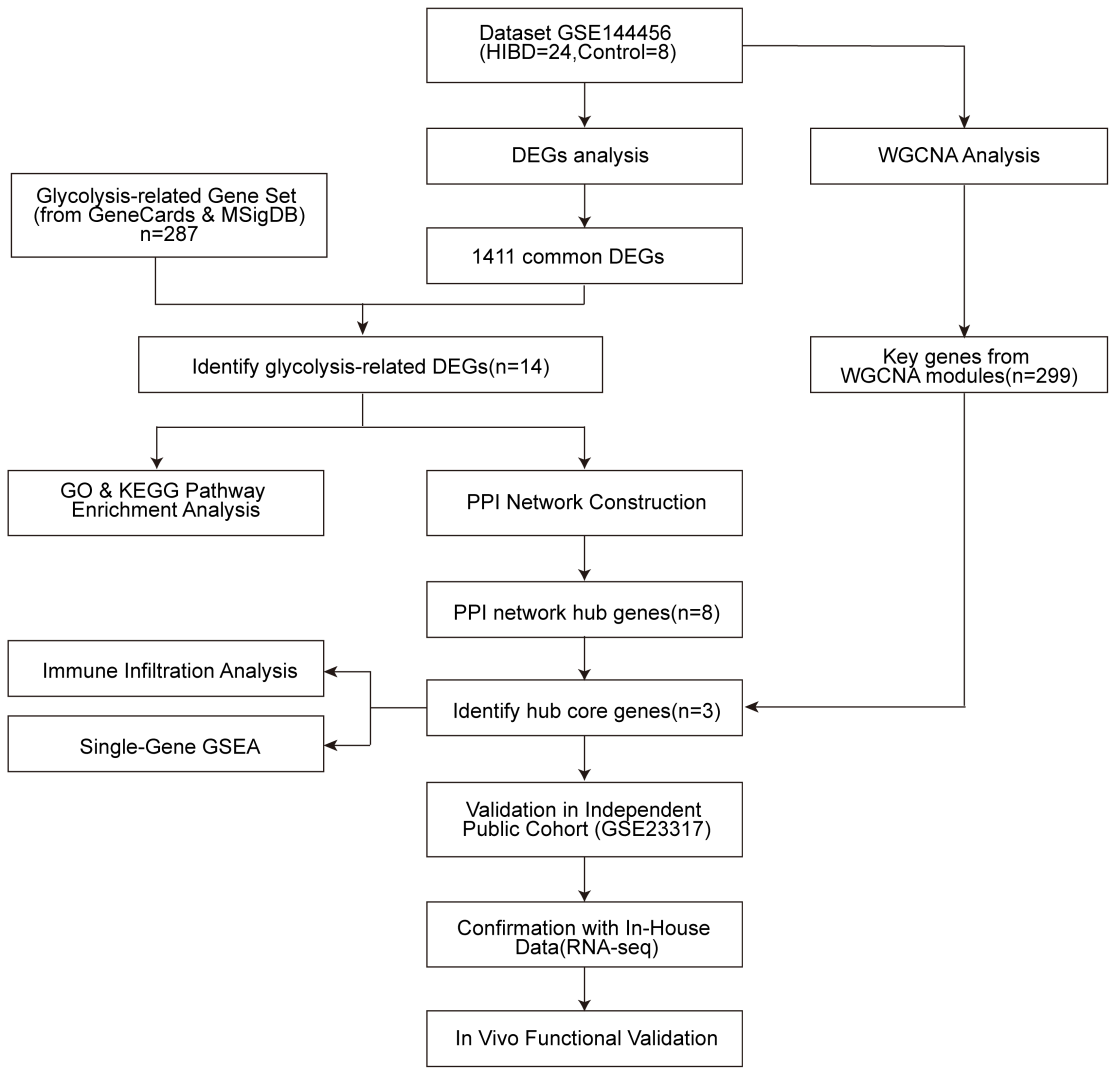
The workflow of this research.

However, the key nodes linking glycolytic reprogramming to neuroinflammation in neonatal HIBD, and the therapeutic potential of targeting them early, are not well defined. To this end, we combined a systems biology approach with *in vivo* validation.​ First, we performed an integrated analysis of transcriptomic datasets​ to identify hub genes central to glycolytic reprogramming. This analysis highlighted *Hk2* as a top candidate. We then confirmed its marked upregulation and correlation with inflammatory markers in a neonatal mouse HIBD model. Finally, to assess the translational potential of this finding, we evaluated whether early pharmacological perturbation of the *Hk2*-centered glycolytic axis with 3-BrPA could mitigate acute injury and confer lasting neurological and structural benefits as assessed by MRI and behavioral tests. The workflow is summarized in **Figure 1**.

## 2 Materials and methods

### 2.1 Bioinformatics data sources and pretreatment

The gene expression profile dataset GSE144456 was downloaded from the Gene Expression Omnibus (GEO) database (http://www.ncbi.nlm.nih.gov/geo/). This dataset is based on the GPL10333 platform and comprises 24 HIBD samples and 8 control samples. The raw expression data were log2-transformed and quantile-normalized using the R package “limma” for subsequent analysis. The independent validation dataset GSE23317, based on the GPL6885 platform, includes 11 HIBD samples and 11 control samples. The expression matrix was subjected to the same preprocessing (**Supplementary Table 1**).

### 2.2 Identification of DEGs and glycolysis-related DEGs

Using the R package “limma”, gene expression profiles at each HIBD time point were compared with those of the control group to identify differentially expressed genes (DEGs), with the criteria of | log2 fold change (log2FC)| > 0.3 and P < 0.05. A relatively inclusive fold-change threshold was chosen to capture broader transcriptional changes in the early phase of HIBD for subsequent network analysis.​ The DEGs from all time points were combined to create a non-redundant DEG set. Glycolysis-related genes retrieved from GeneCards database (https://www.genecards.org/, relevance score > 5) and the MSigDB database (https://www.gsea-msigdb.org/gsea/msigdb) were combined by union to construct a glycolysis-related gene set (n = 287; see **Supplementary Data Sheet 1**). This gene set was then intersected with the aforementioned DEGs to obtain glycolysis-related DEGs for subsequent analysis.

### 2.3 Functional enrichment and network analysis

For the identified glycolysis-related DEGs, Gene Ontology (GO) functional annotation and Kyoto Encyclopedia of Genes and Genomes (KEGG) pathway enrichment analysis were performed using the R package “clusterProfiler” with a significance threshold of P < 0.05. The differentially expressed genes were submitted to the GeneMANIA web tool (https://genemania.org/) to construct a functional interaction network, and to the STRING database (https://string-db.org/) to construct a protein-protein interaction network. Hub genes were identified using the CytoHubba plugin in Cytoscape (version 3.7.2) based on node degree values within the maximal connected subgraph.

### 2.4 Weighted gene co-expression network

A weighted gene co-expression network was constructed using the “weighted gene co-expression network analysis (WGCNA)” R package. The optimal soft-thresholding power (β=7) was selected to achieve a scale-free topology (scale-free R² > 0.85). Genes were clustered into modules using dynamic tree cutting (minModuleSize=50). Module-trait relationships were calculated by correlating module eigengenes with the HIBD phenotype. Key HIBD-associated modules (|r|> 0.7, P < 0.05) were selected.​ For genes within key modules, those with Module Membership (MM) > 0.6 and Gene Significance (GS) for the HIBD phenotype > 0.3 were defined as module hub genes. The final candidate hub genes were obtained by intersecting the top-ranked genes from the protein-protein interaction (PPI) network (MCC algorithm) with the WGCNA module hub genes.​ This intersection strategy was employed to enhance robustness, prioritizing genes central to both physical interaction networks and co-expression modules associated with HIBD, thereby mitigating potential bias from any single network inference method.

### 2.5 Immune infiltration analysis

To characterize the immune microenvironment following HIBD, we applied the CIBERSORT (Cell-type Identification By Estimating Relative Subsets Of RNA Transcripts) deconvolution algorithm (25, 26) to estimate the relative proportions of 25 immune cell subsets from bulk tissue transcriptome data. The complete list of cell type-specific marker genes used for deconvolution was derived from reference (27) and is provided as **Supplementary Data Sheet 2**.

### 2.6 Single-gene gene set enrichment analysis

To investigate the biological pathways associated with the hub genes (*Egfr*, *Hk2*, and *Vegfa*), we performed Gene Set Enrichment Analysis (GSEA) based on the expression level of individual genes. All samples in the GSE144456 dataset were stratified into a “High expression group” and a “Low expression group” according to the median expression of the target hub gene (e.g., *Egfr*), and the log2FC of all genes between the high and low expression groups was calculated. All genes were then ranked in descending order according to their log2FC values to generate a ranked gene list. Enrichment analysis was performed on this ranked gene list using the GSEA function implemented in the R package clusterProfiler, with the Reactome pathway database as the reference gene set. The number of permutations for the analysis was set to 1,000. Pathways with a nominal P < 0.05 were defined as significantly enriched under the context of high or low expression of the corresponding hub gene.

### 2.7 Core gene validation and independent dataset analysis

To validate the differential expression of the core genes in the independent dataset GSE23317, its normalized expression profile was downloaded from the GEO database. The Log2FC for “HIBD *vs*. sham” was calculated, and the P values were corrected using Empirical Bayesian adjustment and the Benjamini-Hochberg method. Genes with P < 0.05 were considered statistically significant. The results are presented as a bar graph showing the mean expression value ± standard error of the mean for each group, with statistical significance indicated.

For the RNA-seq data obtained 24 hours after modeling from the HIBD animal model established by our research group (sham/control, 3/3 per group) (the raw expression matrix is provided in **Supplementary Data Sheet 3**), genes with |log2FC| > 0.5 and P < 0.05 were defined as differentially expressed. These genes were classified into three categories: significantly upregulated, significantly downregulated, and not significantly changed. A volcano plot was generated using the ggplot2 package, and selected core genes were labeled using the ggrepel package.

### 2.8 *In vivo* animal model and drug intervention

Animals were randomly assigned to experimental groups using a random number table. The investigators performing behavioral tests, histological assessments, and image/band quantification were blinded to the group allocation during data acquisition and analysis. The laboratory animals used in this study were housed in a specific pathogen-free animal facility. The housing environment was maintained at a temperature of 22 ± 2 °C, a relative humidity of 50%–70%, and a 12-hour light/dark cycle, with ad libitum access to standard chow and drinking water. All animal experiments were conducted in accordance with the Replacement, Reduction, and Refinement (3R) principles, with efforts made to minimize animal suffering and the number of animals used. Pups of both sexes were used and randomly distributed across groups. Given the exploratory nature of this study and the focus on initial proof-of-concept, data from both sexes were pooled for analysis, as no sex-specific effects were hypothesized *a priori*. Future studies will be designed to explicitly address sex differences. The experimental protocol was approved by the Institutional Animal Ethics Committee (Approval No.: 2024-388-01).

Neonatal C57BL/6 mice at postnatal day 9 (P9, body weight range 5 ± 0.3 g, both sexes) were used in this study. A neonatal hypoxic-ischemic brain damage model was established following the modified Rice-Vannucci method. The procedure was briefly as follows: under isoflurane inhalation anesthesia (induction: 3%–4% in room air; maintenance: 1.5%–2% in room air; flow rate: 0.4 L/min; delivered via a small animal anesthesia machine [RWD Life Science, Shenzhen, China]), a midline cervical incision was made in each mouse, and the right common carotid artery was isolated and permanently ligated. After a 1-hour recovery period, the mice were placed in a sealed hypoxic chamber containing a gas mixture of 10% oxygen and 90% nitrogen and subjected to continuous hypoxia at 37 °C for 1 hour. Mice in the Sham group underwent cervical incision and vessel isolation only, without ligation or hypoxic exposure. Following successful model establishment, HIBD pups were randomly divided into three groups: Sham + Vehicle group, HIBD + Vehicle group, and HIBD + 3-Bromopyruvate treatment group. 3-BrPA (MedChemExpress, HY-19992) or an equal volume of vehicle was administered by intraperitoneal injection 1 hour after model induction. This dose (1 mg/kg) was determined in pilot experiments to be the optimal neuroprotective dose and was applied as the intervention regimen in all subsequent mechanistic and behavioral experiments.

### 2.9 Experimental design and animal grouping

Animal experiments were conducted in two phases. In the initial validation phase, Sham and HIBD groups (n=6 each) were used. At 24 h post-injury, RNA-seq (n=3/group), RT-qPCR and Western blot for HK2 (n=6/group), and TTC staining (n=3/group) were performed to confirm *Hk2* upregulation and model establishment. In the second phase, we first performed double immunofluorescence co-staining on brain sections from Sham and HIBD mice (n=4 per group) at 24 h post-injury to determine the cellular localization of HK2.​ Subsequently, to evaluate the therapeutic potential of targeting HK2, a dose-response study was conducted with Sham+Vehicle, HIBD+Vehicle, and HIBD + 3-BrPA at 0.5, 1, and 2 mg/kg (n=10/group). T2-weighted MRI at 24 h identified 1 mg/kg as the optimal dose. A separate cohort was then assigned to Sham+Vehicle, HIBD+Vehicle, and HIBD + 3-BrPA (1 mg/kg) groups for comprehensive endpoint analyses: lactate assay (n=6/group), RT-qPCR for pro-inflammatory cytokines (n=4/group), Western blot for HK2 and polarization markers (n=4/group), Iba1 immunofluorescence (n=3/group), and longitudinal MRI and behavioral assessment (n=10/group). Randomization and blinding were applied throughout.

### 2.10 Histological and imaging assessment

#### 2.10.1 2,3,5-triphenyltetrazolium chloride staining

To evaluate whether the injury model was successfully established, 2,3,5-triphenyltetrazolium chloride (TTC) staining was performed in a subset of mice 24 h after injury. Mice were deeply anesthetized with isoflurane (5% in room air; flow rate: 0.4 L/min) and then euthanized by decapitation. The whole brain was rapidly removed and frozen at −20 °C for 20 min. The brain was then sectioned coronally into four consecutive thick slices using a blade. The slices were immersed in 2% TTC solution prepared in phosphate-buffered saline and incubated at 37 °C for 20 min in the dark. After staining, the slices were photographed.

#### 2.10.2 Magnetic resonance imaging

To noninvasively and accurately quantify both acute and long-term brain injury, T2-weighted magnetic resonance imaging (MRI) scans were performed on mice at 24 h and 30 days post-injury. Scanning was conducted on a 4.7T small-animal MRI system (MR Solutions, Guildford, Surrey, United Kingdom). Mice were anesthetized with continuous isoflurane inhalation and secured in a dedicated holder. During scanning, mice were anesthetized with inhaled isoflurane (induction: 3%–4%; maintenance: 1.5%–2% in room air; flow rate: 0.4 L/min) delivered via the same small animal anesthesia system. T2-weighted images were acquired using a mouse head quadrature coil.

The scanning sequence was fast spin echo with the following parameters: repetition time = 3000 ms; echo time = 51 ms; echo train length = 7; averages = 4; matrix = 256 × 238; field of view = 25 × 25 mm²; slice thickness = 0.50 mm. The total scanning time was 7 min.

The acquired DICOM images were imported into RadiAnt DICOM Viewer (version 2022.1.1). The infarct area on each slice was manually delineated by an observer, and the total infarct volume was calculated and expressed as a percentage of the volume of the contralateral hemisphere.

### 2.11 Molecular biological analysis

#### 2.11.1 Quantitative real-time polymerase chain reaction

To measure gene and protein expression levels, the injured ipsilateral hemisphere was collected 24 h after model induction. Total RNA was extracted using TRIzol reagent (Life Technologies). RNA concentration and purity were measured using a nucleic acid spectrophotometer, and samples with A260/A280 ratios between 1.8 and 2.2 were used for subsequent experiments. Subsequently, 1 µg of total RNA was reverse-transcribed into 20 µL cDNA using a reverse transcription kit (Vazyme). β-actin was used as the internal reference gene. The relative mRNA expression levels of target genes (*Hk2*, *Tnf*, *Il1b*, and *Il6*) were measured using RT-qPCR. The amplification reaction mixture consisted of 2 µL cDNA template, gene-specific primers (sequences shown in **Table 1**), SYBR PCR Master Mix and reactions were performed on a real-time PCR system. The sequences of all primers used are listed in **Table 1**. Relative gene expression levels were calculated using the 2^−ΔΔCt^ method.

**Table 1:** Primers used for qRT-PCR.

Gene	Forward sequence (5’ to 3’)	Reverse sequence (5’ to 3’)
Tnf	TATGGCTCAGGGTCCAACTC	GGAAAGCCCATTTGAGTCCT
Il1b	TGCCACCTTTGACAGTGATG	TGATGTGCTGCTGCGAGATT
Il6	GGAGTGGCTAAGGACCAAGAC	CATAACGCACTAGGTTTGCCG
Hk2	TGATCGCCTGCTTATTCACGG	AACCGCCTAGAAATCTCCAGA
β-actin	ACGGCCAGGTCATCACTATTG	AGAGGTCTTTACGGATGTCAACGT

#### 2.11.2 Western blot

For protein expression analysis, total protein was extracted from tissue using RIPA lysis buffer, and protein concentrations were determined using the Bicinchoninic Acid assay. Equal amounts of protein were separated by SDS-PAGE and subsequently transferred onto polyvinylidene fluoride membranes. After blocking with 5% non-fat milk for 2 h at room temperature, the membranes were incubated overnight at 4 °C with the following primary antibodies: anti-HK2 (1:1000, Cell Signaling Technology, 2867), anti-inducible nitric oxide synthase (iNOS) (1:1000, HuaAn Biotechnology, ER1706-89), anti-arginase 1 (ARG1) (1:1000, HuaAn Biotechnology, ET1605-8), and anti-β-actin (1:5000, Cell Signaling Technology, 4970) as a loading control. After washing with Tris-Buffered Saline with Tween 20, the membranes were incubated with the corresponding horseradish peroxidase-conjugated secondary antibodies for 1 h at room temperature.

Protein bands were visualized using an enhanced chemiluminescence detection kit, and signals were captured using a chemiluminescence imaging system. Band intensities were quantified using ImageJ software, and protein expression levels were normalized to β-actin.

#### 2.11.3 Immunofluorescence staining

Double immunofluorescence staining was conducted on brain sections from Sham and HIBD mice at 24 hours post-injury. Sections were blocked with PBS containing 10% normal donkey serum and 0.3% Triton X-100 for 1 h at room temperature, followed by incubation with the following primary antibody pairs at 4 °C overnight: mouse anti-HK2 (1:375, Proteintech, 66974-1-Ig) with rabbit anti-Iba1 (1:200, Abcam, ab178846)​ for microglia; mouse anti-HK2 with rabbit anti-NeuN (1:50, MCE, HY-P80241)​ for neurons; and mouse anti-HK2 with rabbit anti-GFAP (1:2500, Abcam, ab7260)​ for astrocytes. After washing, sections were incubated with the corresponding secondary antibody mixture: Goat Anti-Mouse IgG (Alexa Fluor 488 conjugate, 1:500, Proteintech, RGAM004) and Goat Anti-Rabbit IgG (Alexa Fluor 555 conjugate, 1:500, Proteintech, RGAR002)​ for 2 h at room temperature in the dark. All sections were counterstained with DAPI, mounted, and imaged using a microscope. To evaluate the effect of 3-BrPA on microglial morphology, brain sections from Sham+Vehicle, HIBD+Vehicle, and HIBD + 3-BrPA mice were processed for single Iba1 immunofluorescence using rabbit anti-Iba1 primary antibody (1:200, Abcam, ab178846)​ following the same procedure.

#### 2.11.4 Lactate assay

To assess glycolytic flux, brain lactate levels were measured 24 hours after HIBD. Cortical tissue from the ipsilateral hemisphere of Sham+Vehicle, HIBD+Vehicle, and HIBD + 3-BrPA mice was homogenized. The lactate concentration in the supernatant was determined using a Lactate Assay Kit (Nanjing Jiancheng Bioengineering Institute, A019-2-1) according to the manufacturer’s instructions. Absorbance was measured at 530 nm, and lactate content was normalized to the total protein concentration and expressed as μmol/mg protein.

### 2.12 Behavioral assessment

#### 2.12.1 Rotarod test

Motor coordination was assessed using the rotarod test on day 30 after injury. An automated rotarod apparatus was used. During testing, mice were placed directly onto the stationary rotating rod facing the direction of rotation. After the test started, the rod accelerated linearly from 4 rpm to 40 rpm within 30 s and then maintained the maximum speed. The latency to fall from the rotating rod onto the platform below was recorded. Each mouse was tested three times, with a minimum 30-min rest interval between trials. The average latency of the three trials was used as the final score. If a mouse did not fall within 300 s after the start of the test (including the acceleration phase), the latency was recorded as 300 s. All tests were conducted in a quiet experimental environment with uniform lighting.

#### 2.12.2 Elevated zero maze test

The elevated zero maze test was performed on day 30 after injury to assess anxiety-like behavior in mice. The maze consisted of a continuous circular platform elevated 50 cm above the floor. The outer diameter was 45 cm and the inner diameter was 33 cm, forming a 6-cm-wide circular track. The track was divided into four equal quadrants: two opposite closed quadrants (with 15-cm-high walls) and two opposite open quadrants (without walls). The experiment was conducted in a quiet, sound-attenuated environment with uniform illumination. At the beginning of the test, each mouse was gently placed in the center of a closed quadrant and allowed to explore freely for 5 min. The entire session was recorded using a camera positioned above the apparatus.

Locomotor parameters were analyzed using video tracking software, including total distance traveled, percentage of time spent in the open quadrants and average velocity.

### 2.13 Statistical analysis

All data are presented as mean ± standard deviation. Statistical analyses were performed using GraphPad Prism 9.0. Comparisons between two groups were performed using the unpaired two−tailed Student’s t−test. Comparisons among multiple groups were conducted using one−way analysis of variance. When the assumption of equal variance was met, Tukey’s *post hoc* test was used for multiple comparisons. Pearson correlation analysis was used to evaluate correlations between variables. P < 0.05 was considered statistically significant.

## 3 Results

### 3.1 Identification of glycolysis-related DEGs in HIBD by bioinformatics analysis

**Figure 2:**
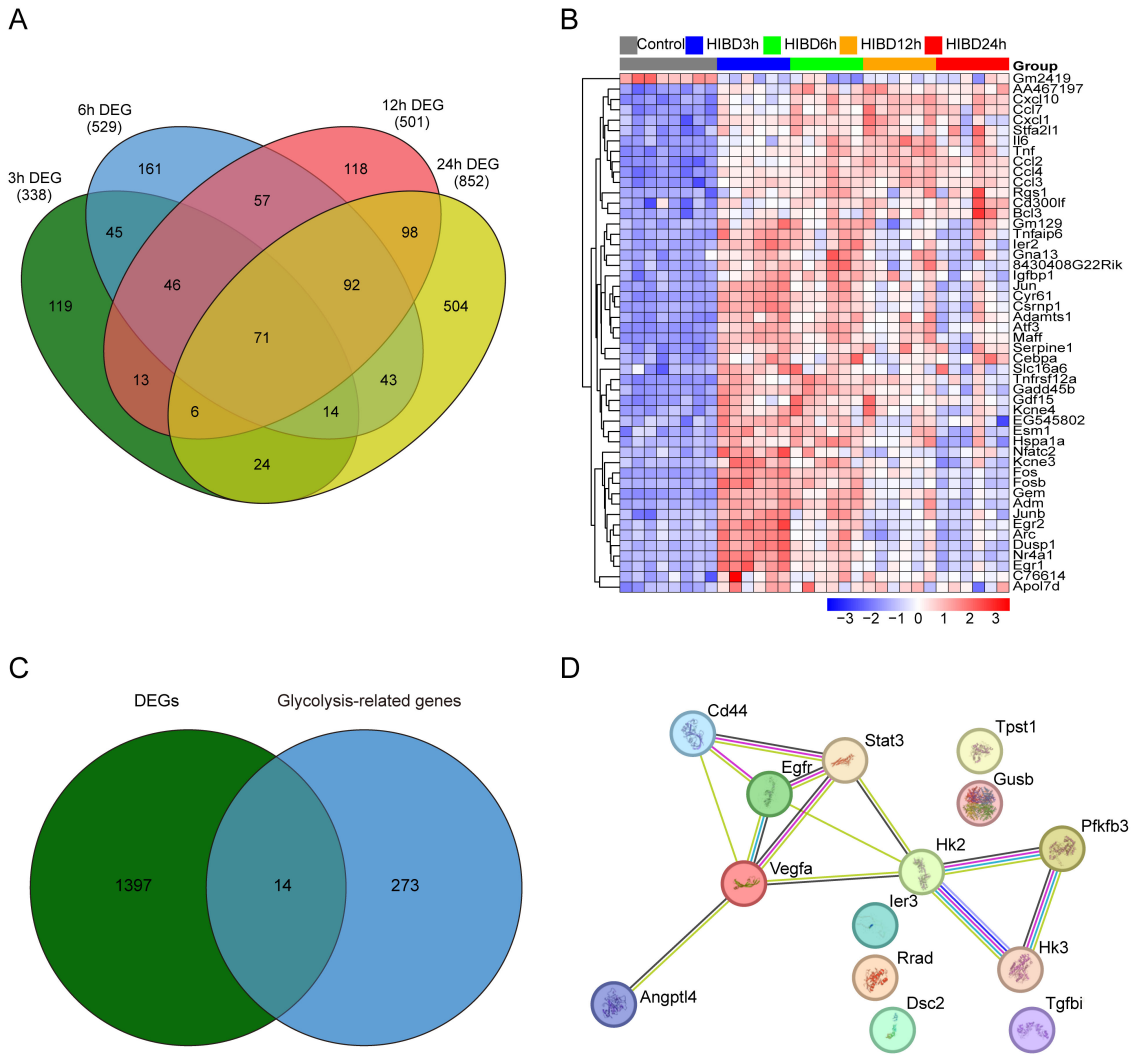
Screening and analysis of glycolysis-related DEGs in HIBD. **(A)** Identification of DEGs at multiple time points post-HIBD; **(B)** Expression patterns of top DEGs across time points; **(C)** Screening of glycolysis-related DEGs; **(D)** PPI network of key glycolysis-related DEGs.

To systematically investigate transcriptomic alterations following HIBD, we first analyzed the GEO dataset GSE144456. By comparing samples at 3, 6, 12, and 24 hours after HIBD with those in the control group, 338, 529, 501, and 852 DEGs were identified, respectively. The union of these datasets yielded 1411 non-redundant DEGs (**Figure 2A**). A heatmap of the top 50 most significantly altered genes demonstrated distinct expression patterns between HIBD samples at different time points and the controls (**Figure 2B**). To further focus on metabolic alterations, the identified DEGs were intersected with a glycolysis-related gene set, resulting in 14 glycolysis-related DEGs, which were selected as candidate targets for subsequent analyses (**Figure 2C**). Analysis via the STRING database revealed a centralized PPI module among these glycolysis-related DEGs (**Figure 2D**).

### 3.2 Functional enrichment and interaction network analysis of glycolysis-related DEGs

**Figure 3:**
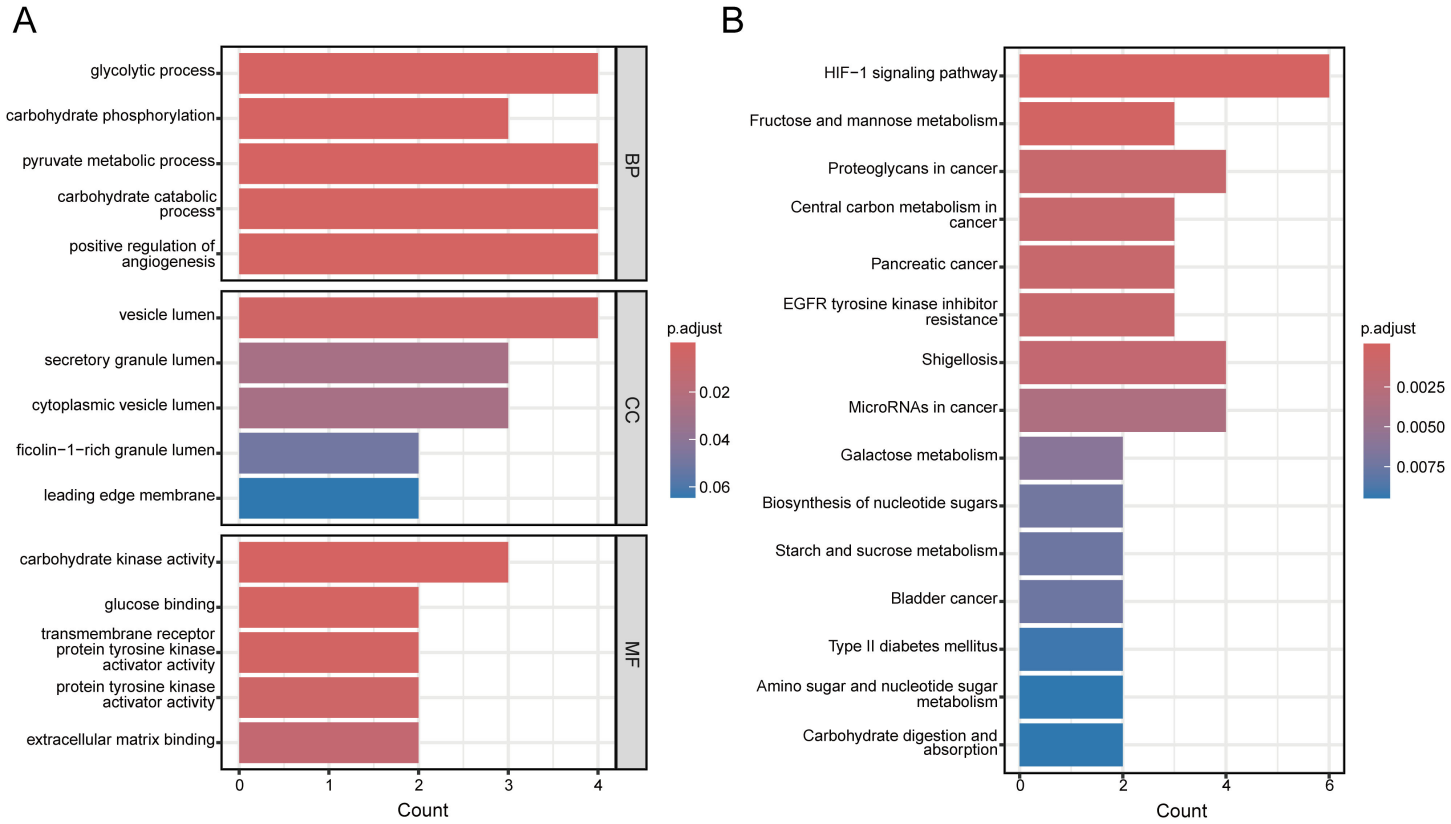
Functional and pathway enrichment analysis of differentially expressed genes. **(A)** GO enrichment analysis (BP, biological process; CC, cellular component; MF, molecular function); **(B)** KEGG pathway enrichment analysis.

Functional annotation was performed for the 14 glycolysis-related DEGs. GO enrichment analysis indicated that these genes were mainly involved in biological processes such as glycolytic process and carbohydrate phosphorylation, and exhibited molecular functions including carbohydrate kinase activity (**Figure 3A**). KEGG pathway analysis revealed that these genes were significantly enriched in the HIF-1 signaling pathway and other metabolism-related pathways such as carbohydrate digestion and absorption (**Figure 3B**).

**Figure 4:**
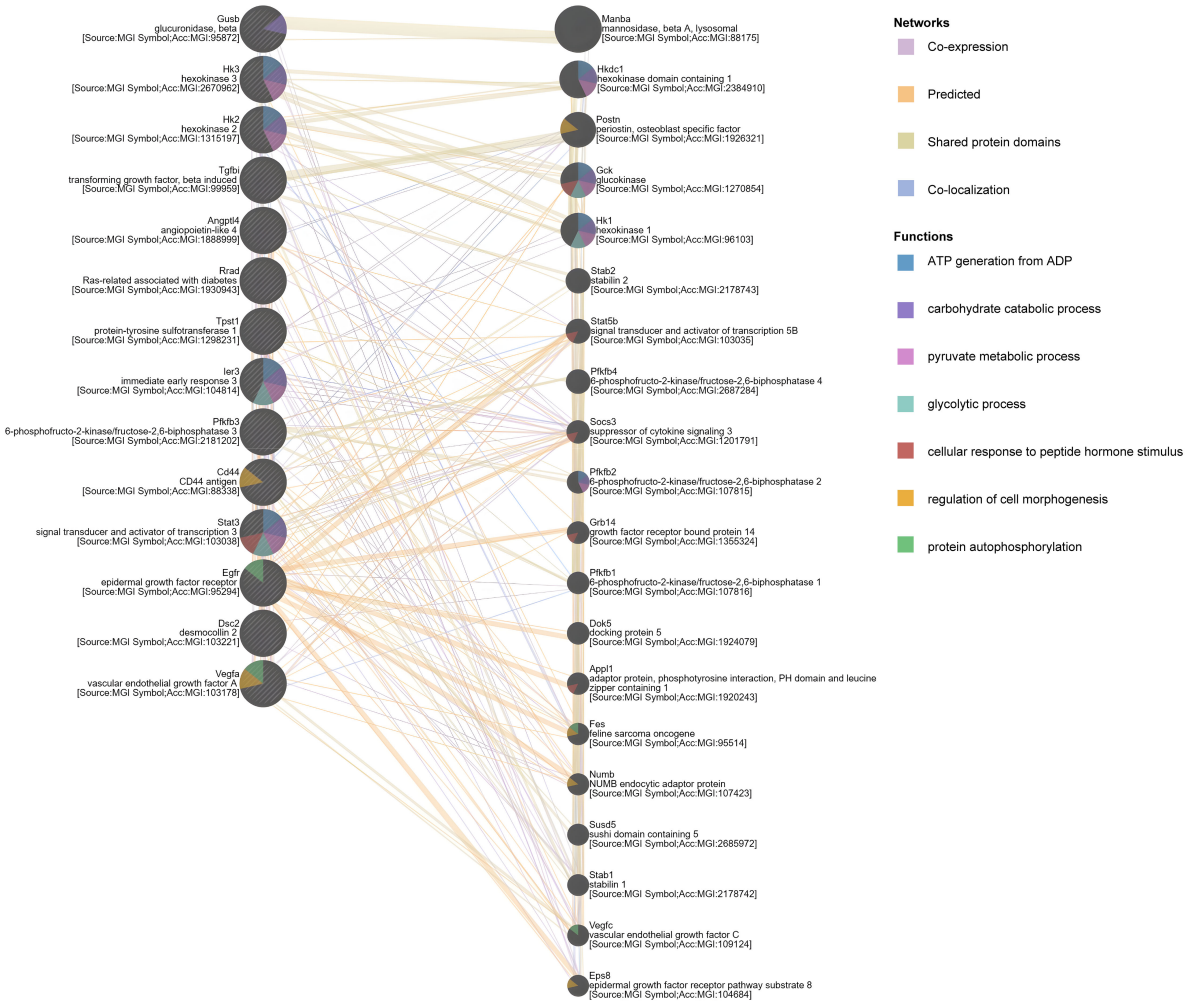
Functional interaction network and enriched biological processes of glycolysis-related DEGs.

**Figure 5:**
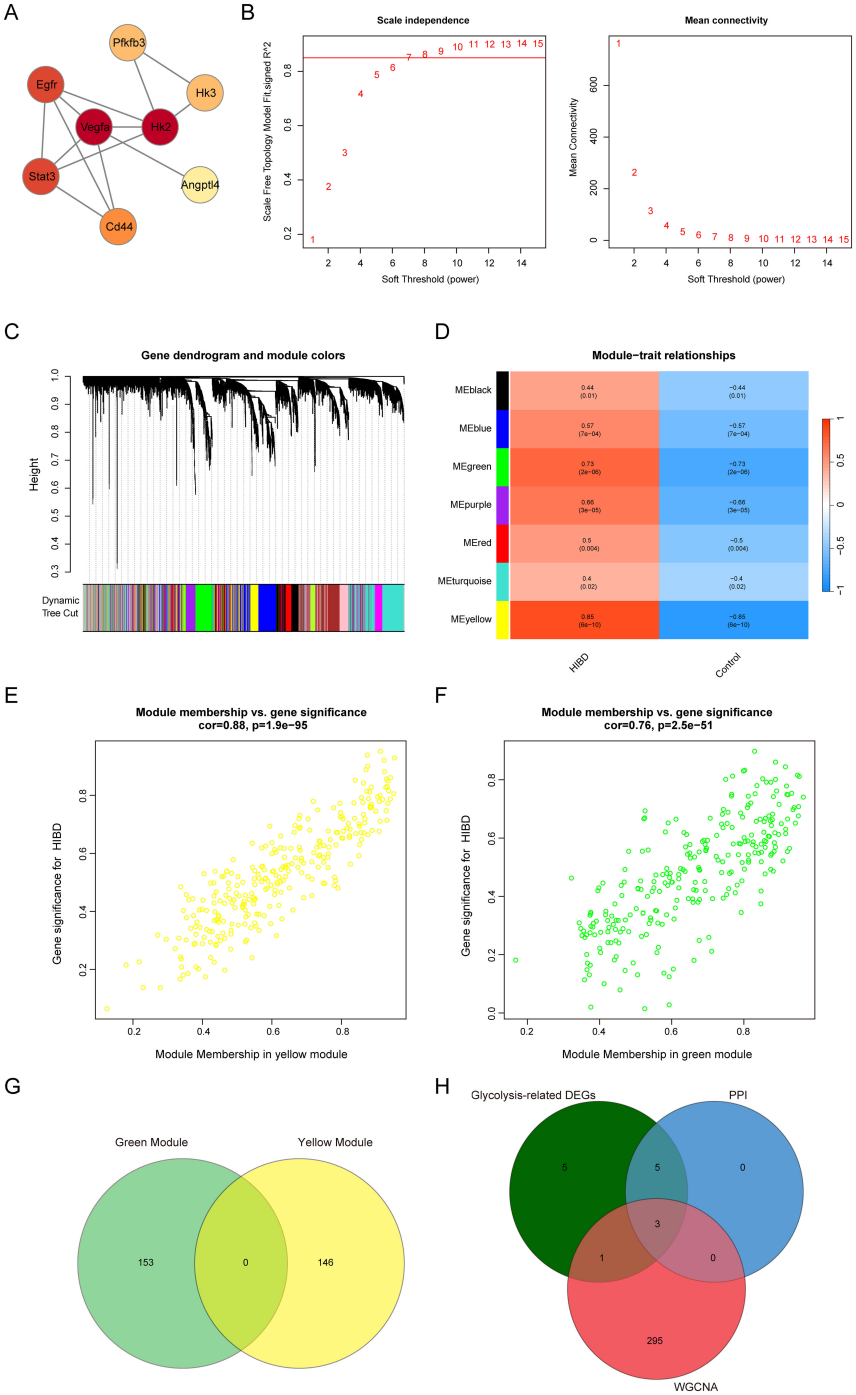
Identification of key regulatory genes via integrated network analysis. **(A)** PPI network highlighting top 8 hub genes identified by cytoHubba. **(B)** Selection of optimal soft-thresholding power for WGCNA. **(C)** Gene clustering dendrogram and module assignment from WGCNA. **(D)** Module–trait correlation analysis in WGCNA. **(E, F)** Analysis of GS *vs*. MM in key WGCNA modules. **(G)** Venn diagram showing the intersection of hub genes from key WGCNA modules. **(H)** Venn diagram identifying the common core genes from PPI and WGCNA analyses.

The functional interaction network constructed by the GeneMANIA online tool, which integrates the 14 core genes with their 20 most frequently associated partners (e.g., Manba, Hkdc1, Hk1), further demonstrated that these genes are strongly associated with biological functions including the glycolytic process and ATP generation (**Figure 4**). To explore coordinated gene regulation patterns, WGCNA was performed to construct a gene co-expression network. Through soft-threshold analysis, β = 7 was determined to be the optimal parameter (scale-free topology fit index R² > 0.85) (**Figure 5B**). A total of 12 gene co-expression modules were identified. To highlight key findings, the clustering tree of seven modules with relatively strong phenotype associations was shown in **Figure 5C**. Further module–trait correlation analysis (**Figure 5D**) revealed that among these seven modules, the green module (r = 0.73, P = 2×10^-6^) and the yellow module (r = 0.85, P = 6×10^-10^) exhibited the strongest positive correlations with the HIBD phenotype and were therefore selected as key modules for subsequent analysis. From these two modules, genes with MM > 0.6 and GS > 0.3 were selected, yielding 299 highly connected module hub genes. (**Figures 5E–G**) To identify core regulatory genes, a protein–protein interaction network based on the 14 genes was constructed, which revealed 8 hub genes as determined by the Degree algorithm using the cytoHubba plugin in Cytoscape (**Figure 5A**). By intersecting these hub genes with the 299 module hub genes obtained from WGCNA, *Egfr*, *Hk2*, and *Vegfa* were ultimately identified as the three common core genes (**Figure 5H**).

### 3.3 Association between core genes and the immune microenvironment and pathway enrichment analysis

**Figure 6:**
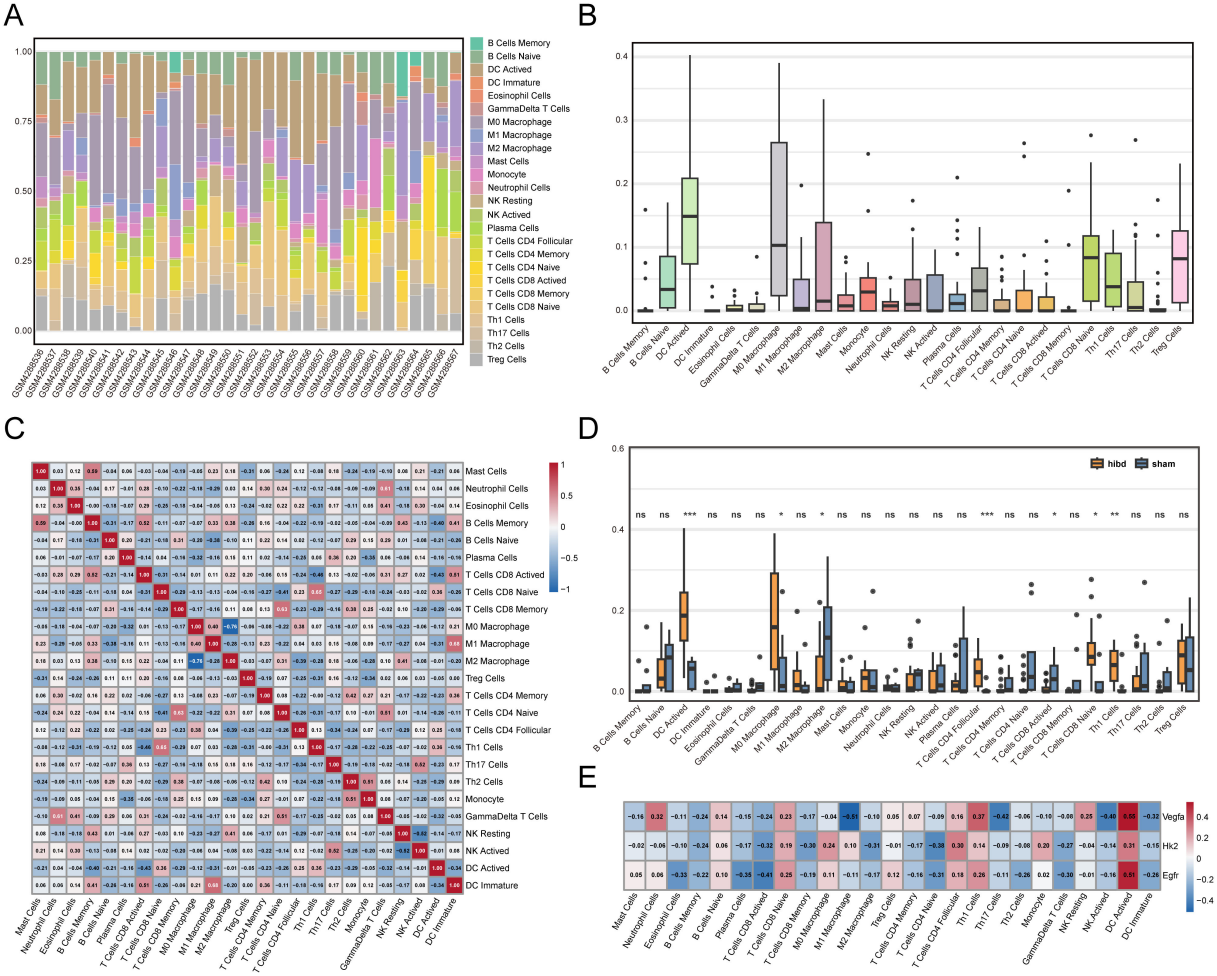
Analysis of immune cell infiltration in HIBD brain tissue. **(A)** Proportional composition of 25 immune cell types across individual samples. **(B)** Relative abundance heatmap of 25 immune cell types across all samples. **(C)** Correlation matrix of immune cell subpopulations. **(D)** Differential infiltration levels of key immune cells (HIBD *vs*. control). **(E)** Correlation between core gene (*Hk2*, *Egfr*, *Vegfa*) expression and immune cell abundance. ns, not significant (P > 0.05), *P <0.05, **P <0.01, ***P <0.001.

To gain insights into the immune landscape following HIBD, we performed transcriptomic deconvolution using CIBERSORT. It should be noted that this approach provides an inferred profile of immune cell abundances based on bulk tissue gene expression. The relative proportions of 25 immune cell types across samples are shown in **Figure 6A**. Differential analysis revealed a shift in immune cell composition in HIBD samples compared to sham controls (**Figures 6B, D**). Specifically, the proportions of activated dendritic cells, Tfh cells, Th1 cells, M0 macrophages, and naive CD8^+^ T cells were significantly increased. In contrast, the proportions of activated CD8^+^ T cells and M2 macrophages were significantly decreased. The proportion of neutrophils remained unchanged. Correlations among these immune cell subsets are shown in **Figure 6C**. We next examined whether the expression of the three candidate hub genes (*Hk2*, *Vegfa*, *Egfr*) was associated with specific immune cell populations (**Figure 6E**). *Hk2* expression showed a significant positive correlation with activated DCs (r = 0.31, P < 0.05) and a negative correlation with M2 macrophages (r = -0.31, P < 0.05). Vegfa expression exhibited a stronger positive correlation with activated DCs (r = 0.55, P < 0.05) and a negative correlation with M1 macrophages (r = -0.51, P < 0.05). *Egfr* expression was negatively correlated with activated CD8^+^ T cells (r= -0.41, P < 0.05) and positively correlated with activated DCs (r = 0.51, P < 0.05). Single-gene GSEA suggested that high expression of *Hk2* was associated with enrichment of pathways related to intracellular transport. The full GSEA results for all three genes are provided in **Supplementary Figure 1**.

### 3.4 Validation of the core gene Hk2 in an independent dataset and an animal model

**Figure 7:**
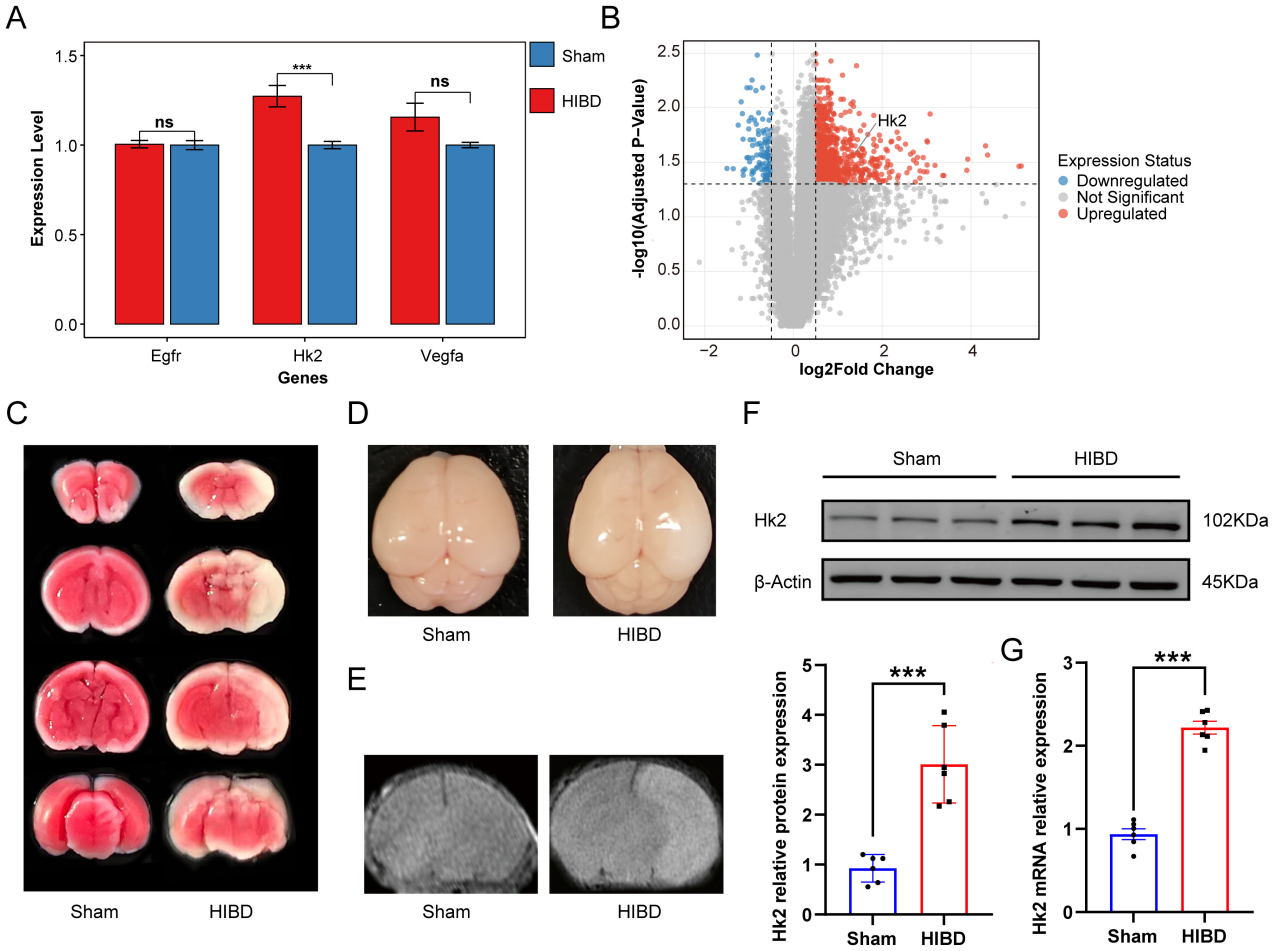
Validation of *Hk2* expression in GSE23317 and in-house transcriptome, and in the HIBD model. **(A)** Validation of core gene (*Hk2*, *Egfr*, *Vegfa*) mRNA expression in the independent GEO dataset GSE23317. **(B–G)** Validation in the 24-hour HIBD model: **(B)** Volcano plot of RNA-seq data; **(C)** Representative TTC-stained brain sections from the indicated groups at 24 h post-HIBD, confirming infarction (n=3/group; qualitative assessment); **(D)** Macroscopic appearance of brain tissue; **(E)** Representative T2-weighted MRI; **(F)** Western blot analysis of HK2 protein level (n=6/group; unpaired two-tailed Student’s t-test, HIBD+Vehicle *vs*. Sham). **(G)** Quantitative RT-qPCR analysis of *Hk2* mRNA level (n=6/group; unpaired two-tailed Student’s t-test; HIBD+Vehicle *vs*. Sham). ns, not significant (P < 0.05), ***P >0.001.

To validate the bioinformatic findings, we examined the independent HIBD dataset GSE23317. Among the three candidate genes, only Hk2 expression was significantly higher in the HIBD group compared to the control group (P < 0.001; **Figure 7A**). We next confirmed this finding in our experimental HIBD mouse model. RNA-sequencing of ipsilateral brain tissue at 24 hours post-injury confirmed the significant upregulation of *Hk2* (log2FC = 1.34, P < 0.05; **Figure 7B**). Successful model establishment was verified by TTC staining and T2-weighted MRI, which revealed clear cerebral infarction (**Figures 7C, E**). Consistent with the transcriptomic data, molecular analyses demonstrated that both HK2 protein (**Figure 7F**) and *Hk2* mRNA (**Figure 7G**) levels were significantly increased in the ipsilateral hemisphere of HIBD mice compared to the Sham controls (both P < 0.001).

### 3.5 HK2 is predominantly upregulated in microglia after HIBD

**Figure 8:**
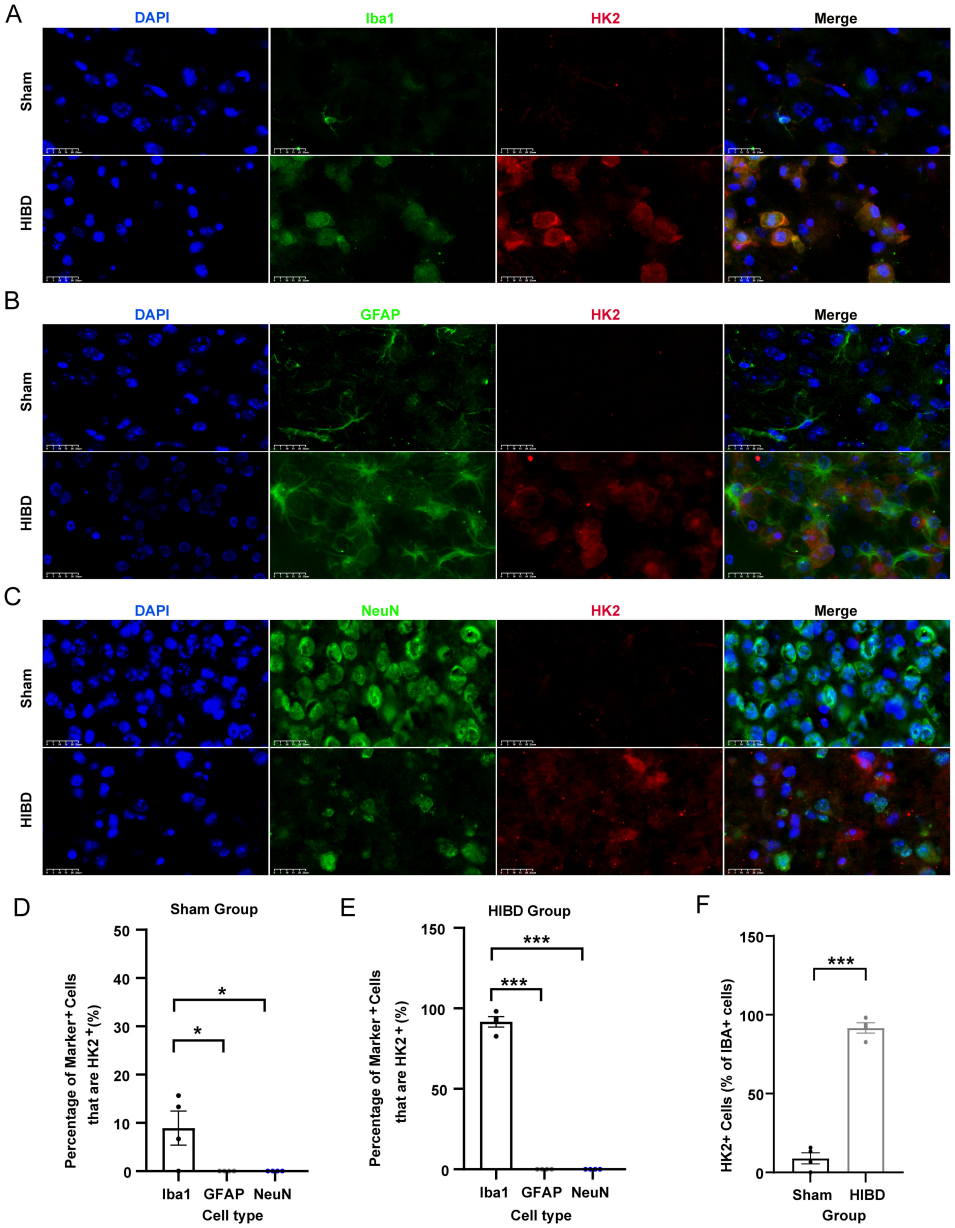
Cellular localization of HK2 in the ipsilateral hemisphere at 24 h post-HIBD. **(A-C)** Representative double immunofluorescence images showing co-localization of HK2 (green) with Iba1 (red, A), GFAP (red, B), or NeuN (red, C) in the peri-infarct region. Nuclei were counterstained with DAPI (blue) (200× magnification; Scale bar = 25 µm). **(D)** Proportion of HK2^+^ cells that were also positive for Iba1, GFAP, or NeuN in the Sham group. **(E)** Proportion of HK2^+^ cells that were also positive for Iba1, GFAP, or NeuN in the HIBD group. **(F)** Comparison of the proportion of HK2^+^ and Iba1^+^ double-positive cells between Sham and HIBD groups. Data are presented as mean ± SD (n=4/group). Statistical analysis: **(D, E)** one-way ANOVA with Tukey’s *post hoc* test for comparisons among three cell types within the same group; **(F)** unpaired two-tailed Student’s t-test for comparison between two groups. *P < 0.05, ***P < 0.001; ns, not significant" (P> 0.05).

Representative double immunofluorescence images revealed substantial co-localization of HK2 with the microglial marker Iba1 in the ipsilateral hemisphere of HIBD mice (**Figure 8A**), but minimal overlap with GFAP (astrocytes) or NeuN (neurons) (**Figures 8B, C**). Quantification within the Sham group showed that, under basal conditions, HK2 was predominantly expressed in Iba1-positive cells, with a significantly higher co-localization rate compared to GFAP or NeuN (both P < 0.05; **Figure 8D**). This predominance was drastically amplified after HIBD, where the proportion of HK2 and Iba1 double-positive cells far exceeded that of cells co-expressing HK2 with GFAP or NeuN (P < 0.001; **Figure 8E**). Moreover, the proportion of HK2-positive microglia (Iba1^+^) was significantly higher in the HIBD group than in the Sham group (P < 0.001; **Figure 8F**).

### 3.6 3-BrPA attenuates acute brain injury in a dose-dependent manner

**Figure 9:**
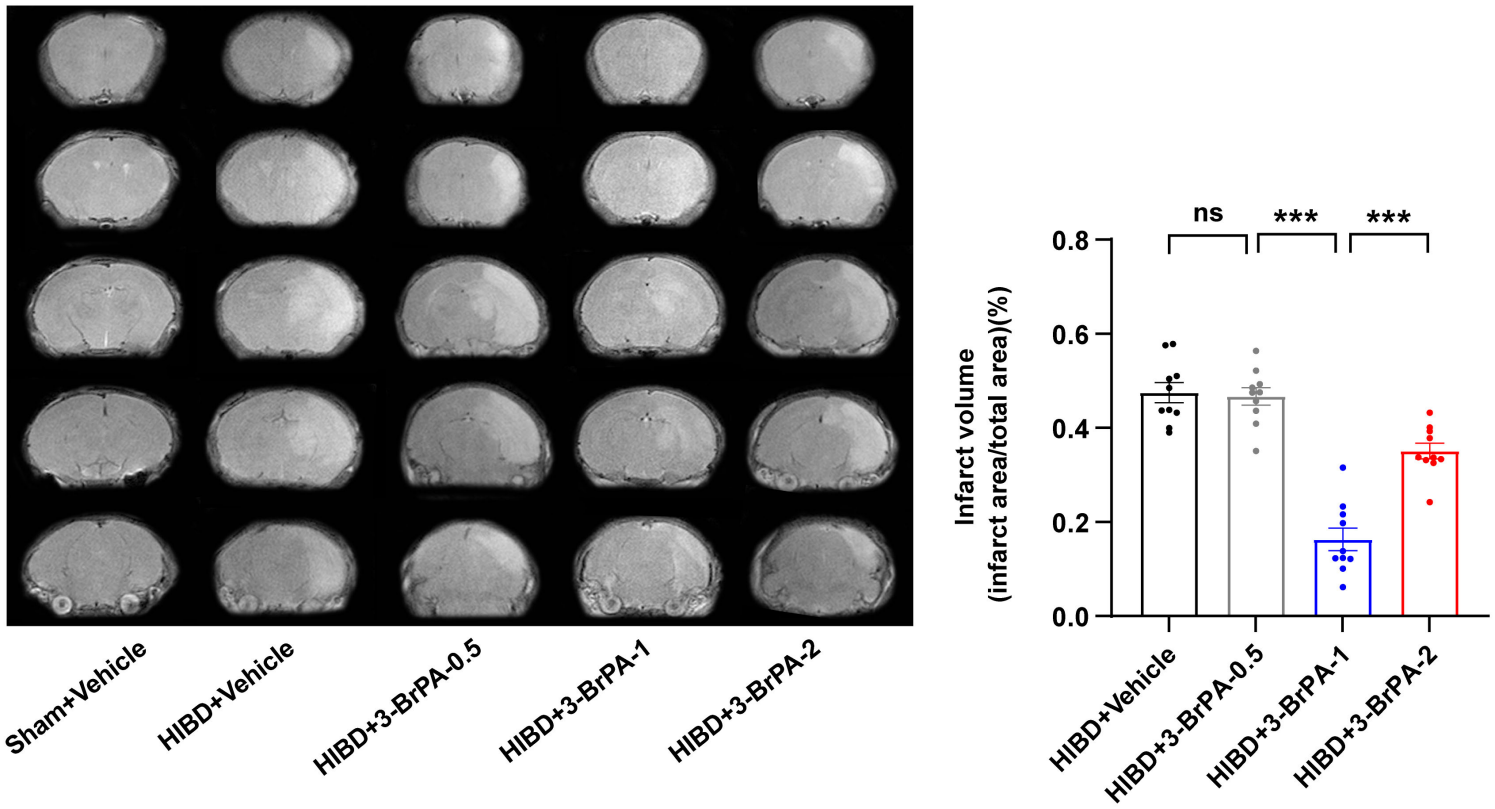
Statistical analysis of cerebral infarction volume at 24 hours post-HIBD after treatment with different doses of 3-BrPA (0.5, 1, 2 mg/kg; n=10/group). Data were analyzed by one-way ANOVA with Tukey’s *post hoc* test. ns, not significant (P < 0.05), ***P >0.001.

Based on the finding that *Hk2* was upregulated in HIBD, we administered 3-BrPA to evaluate its therapeutic effect. A dose-response assessment using T2-weighted MRI at 24 hours post-injury showed that treatment with 0.5, 1, or 2 mg/kg 3-BrPA reduced brain infarct volume in a dose-dependent manner (**Figure 9**). The reduction was most pronounced at the 1 mg/kg​ dose compared to the HIBD + Vehicle group (P < 0.001). Based on these results, 1 mg/kg​ was selected as the optimal dose for all subsequent experiments.

### 3.7 Effects of optimal-dose 3-BrPA on HK2, glycolytic flux, and neuroinflammatory markers

**Figure 10:**
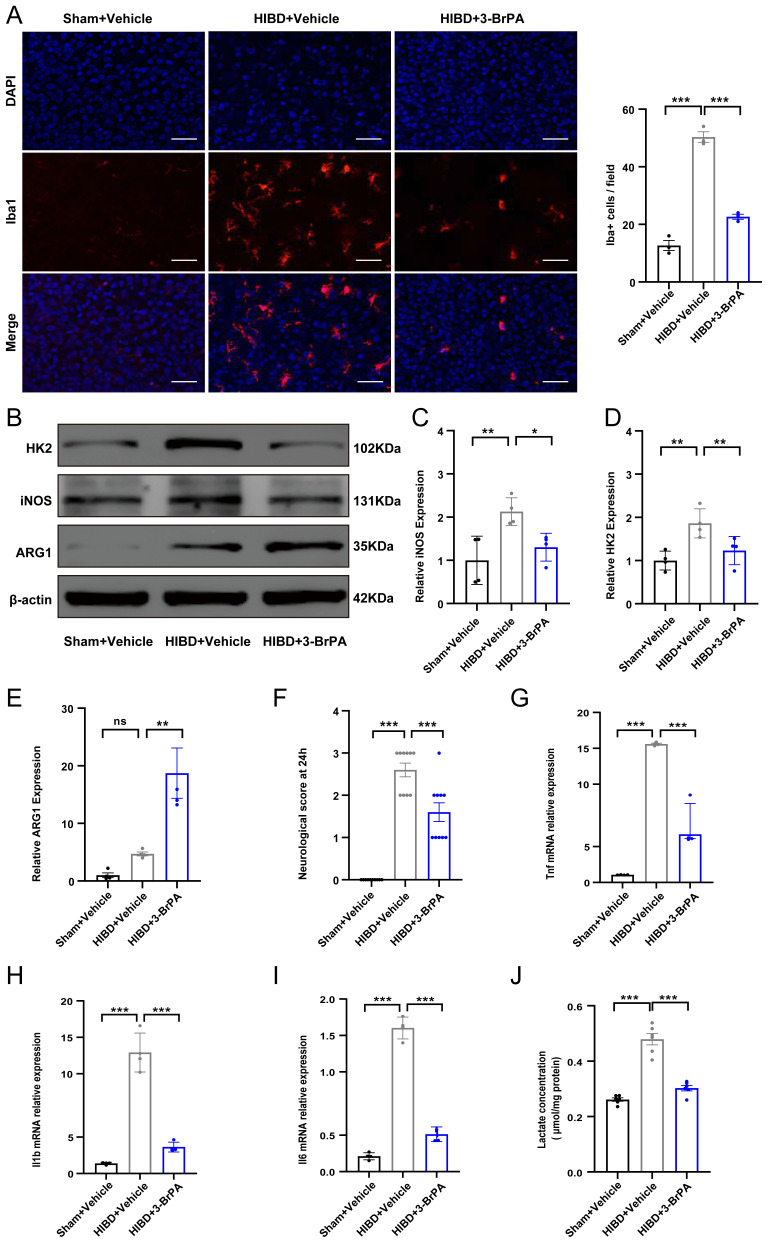
Effects of optimal-dose 3-BrPA (1 mg/kg) on HK2, lactate, neuroinflammatory markers, and neurological function at 24 h post-HIBD. **(A)** Representative immunofluorescence images and quantitative analysis of Iba1^+^ microglia in the ipsilateral cortex. Images are from the peri-infarct region (n=3 animals/group; 200× magnification; scale bar=50 µm). **(B)** Representative Western blot bands of microglial/macrophage polarization markers (iNOS, ARG1) and HK2 at 24 hours post-injury. **(C–E)** Protein levels of iNOS **(C)**, HK2 **(D)**, and ARG1 **(E)** normalized to β-actin (n=4/group). **(F)** Neurological deficit score (Longa score) (n=10/group). **(G–I)** mRNA levels of *Tnf* **(G)**, *Il1b* **(H)**, and *Il6* **(I)** (n=4/group). **(J)** Lactate levels in the ipsilateral hemisphere (n=6/group). Data **(A, C–J)** were analyzed by one-way ANOVA with Tukey’s *post hoc* test. ns not significant (P > 0.05),*P < 0.05,**P < 0.01,***P < 0.001.

3-BrPA (1 mg/kg) altered the morphology of Iba1^+^ microglia in HIBD mice (**Figure 10A**). Iba1^+^ microglia exhibited enlarged cell bodies and a significantly increased density in the ipsilateral hemisphere of the HIBD + Vehicle group compared to the Sham group (P < 0.001). 3-BrPA treatment attenuated these changes, resulting in a significantly lower microglial density compared to the HIBD + Vehicle group (P < 0.001). The mRNA levels of *Tnf*, *Il1b*, and *Il6* were lower in the 3-BrPA group than in the HIBD + Vehicle group (all P < 0.001; **Figures 10G–I**). The protein level of iNOS was decreased (P < 0.05; **Figures 10B, C**), while the protein level of ARG1 was increased (P < 0.01; **Figures 10B, E**) in 3-BrPA-treated mice compared to the HIBD + Vehicle group. HK2 protein levels, which were increased after HIBD, were lower in 3-BrPA-treated mice compared to the HIBD + Vehicle group (P < 0.01; **Figures 10B, D**). Lactate levels in the ipsilateral hemisphere were also reduced by 3-BrPA treatment (P < 0.001 *vs*. HIBD + Vehicle; **Figure 10J**). The neurological deficit score (Longa score) at 24 hours was higher in the 3-BrPA group than in the HIBD + Vehicle group (P < 0.001; **Figure 10F**).

### 3.8 Long-term outcomes assessed by MRI and behavioral tests after early 3-BrPA intervention

**Figure 11:**
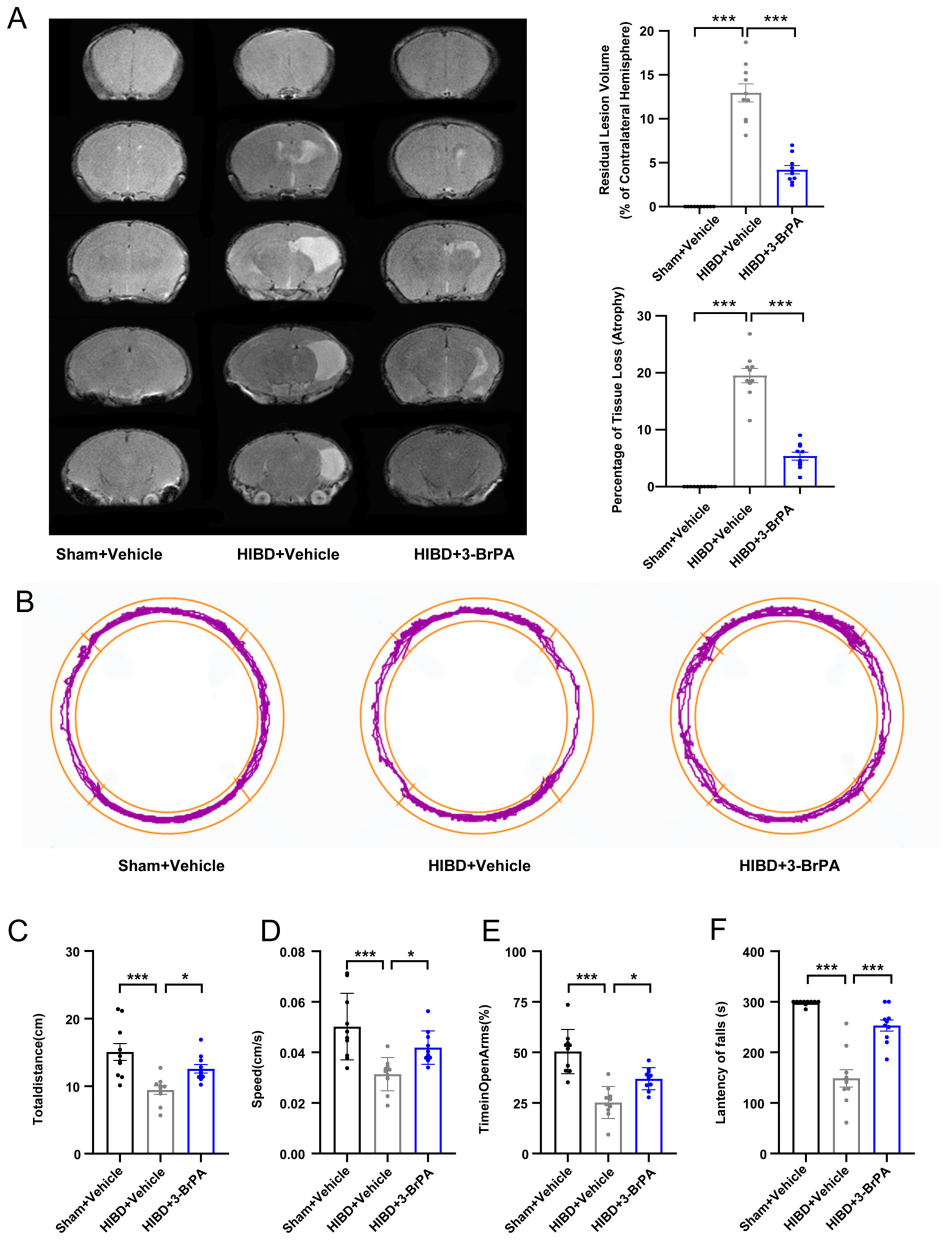
Effects of a single early intervention with 3-BrPA (1 mg/kg) on long-term structural damage and behavioral functions at 30 days post-HIBD. **(A)** Quantification of residual lesion and tissue atrophy volume by T2-weighted MRI (n=10/group). **(B)** Representative movement traces in the elevated zero maze. **(C-E)** Total distance **(C)**, average velocity **(D)**, and time in open quadrants **(E)** in the elevated zero maze (n=10/group). **(F)** Latency to fall in the rotarod test (n=10/group). Data **(A, C–F)** were analyzed by one-way ANOVA with Tukey’s *post hoc* test. *P < 0.05, ***P < 0.001.

The sustained effects of 3-BrPA were evaluated at 30 days post-injury. T2-weighted MRI revealed that the percentages of residual lesion volume and tissue atrophy volume were both significantly lower in the 3-BrPA-treated group compared to the HIBD + Vehicle group (P < 0.001; **Figure 11A**). Behavioral assessments demonstrated long-term functional recovery. Representative movement traces from the elevated zero maze are shown in **Figure 11B**. Quantitative analysis of this test showed that 3-BrPA treatment increased the total distance traveled (P < 0.05 *vs*. HIBD + Vehicle; **Figure 11C**), the average velocity (P < 0.05 *vs*. HIBD + Vehicle; **Figure 11D**), and the percentage of time spent in the open quadrants (P < 0.05 *vs*. HIBD + Vehicle; **Figure 11E**). In the rotarod test, mice treated with 3-BrPA exhibited a significantly longer latency to fall (P < 0.001 *vs*. HIBD + Vehicle; **Figure 11F**).

## 4 Discussion

HIBD is a leading cause of neonatal neurological dysfunction (5, 28). The narrow therapeutic window and partial inefficacy of therapeutic hypothermia highlight the urgent need to explore new therapeutic targets (29, 30). The energy crisis triggered by HIBD initiates a series of pathophysiological events, including glycolytic reprogramming, which is closely coupled with neuroinflammation (2, 7, 11, 31, 32). In this study, by integrating multi-timepoint transcriptomics, bioinformatics, and *in vivo* experimental validation, we systematically investigated the glycolytic reprogramming following HIBD, identified *Hk2* as a key candidate gene associated with both metabolic dysregulation and neuroinflammation, and demonstrated that early intervention with the compound 3-BrPA (33), an inhibitor of hexokinase activity, is associated with neuroprotective effects from the acute to the long-term phase. This provides new experimental insights for potential interventions in HIBD from the perspective of the metabolism-immune axis (34, 35).

We first performed a systematic analysis of the dynamic transcriptomic changes after HIBD. Differential expression analysis combined with a glycolysis-related gene set identified 14 consistently upregulated glycolysis-related DEGs in HIBD. Functional enrichment analysis showed that these genes were significantly enriched in the glycolytic process and the HIF-1 signaling pathway, consistent with hypoxia being a core driver of this metabolic shift (36–38). To identify key regulatory nodes from the network, we employed a cross-validation strategy integrating PPI network analysis and weighted gene WGCNA. This approach, by converging genes central to both physical interactions and co-expression patterns, was used to enhance the robustness of hub gene discovery. Ultimately, *Hk2*, *Egfr*, and *Vegfa* were identified as candidate hub genes. The upregulation of *Hk2* was consistently validated at both the mRNA and protein levels in an independent dataset (GSE23317) and in our established HIBD animal model. As the rate-limiting enzyme of glycolysis, the upregulation of *Hk2* is indicative of a shift in glucose metabolism towards “aerobic glycolysis”, which may contribute to lactate accumulation and suppression of oxidative phosphorylation (39), thereby potentially exacerbating the post-ischemic metabolic crisis.

An important finding of this study is the association between *Hk2* upregulation and the remodeling of the neuroinflammatory microenvironment after HIBD. Immune infiltration analysis suggested a shift in immune cell composition towards a pro-inflammatory phenotype post-HIBD, with *Hk2* expression showing a positive correlation with activated dendritic cells and a negative correlation with M2 macrophages. This aligns with the broader concept of immunometabolic coupling, whereby the metabolic state of immune cells is linked to their functional phenotype (40). Notably, our double immunofluorescence staining directly demonstrated that at 24 hours post-HIBD, the upregulated HK2 protein was predominantly co-localized with Iba1-positive microglia, with minimal co-localization observed with neurons (NeuN) or astrocytes (GFAP)​. This cell type-specific upregulation at the protein level extends previous reports to the context of neonatal HIBD (41). This finding provides direct cellular-level evidence for understanding the link between metabolic reprogramming and functional activation of microglia after HIBD (22, 42).

To experimentally probe this association, and considering both the embryonic lethality associated with *Hk2* knockout (43, 44) and the exacerbation of neuroinflammation upon its complete loss observed in Alzheimer’s disease models (21, 45), we employed the widely used pharmacological *Hk2* inhibitor 3-BrPA (46–49). In the field of cerebral ischemia research, several studies, including those by Swanson’s team, classify 3-BrPA alongside lonidamine (LND) as pharmacological agents that target *Hk2*-mediated glycolysis, distinguishing them from the broader hexokinase inhibitor 2-DG. This “preferential effect” is often discussed in the context of cell type-specific expression: under basal conditions, myeloid cells like microglia express higher levels of *Hk2*, while neurons primarily rely on *Hk1 (*50–52). Therefore, it is proposed that agents like 3-BrPA may have a more limited impact on neurons, allowing for a greater relative effect on immune cell metabolism (18). In the present study, early administration of 3-BrPA was associated with an attenuation of the HIBD-induced upregulation of HK2 protein. More importantly, 3-BrPA treatment was also associated with a significant reduction in lactate accumulation in the injured brain tissue, providing key functional evidence that 3-BrPA can suppress glycolytic flux *in vivo*​. Concurrently, 3-BrPA intervention was associated with the downregulation of pro-inflammatory cytokine (*Tnf*, *Il1b*, *Il6*) mRNA expression and modulation of microglial/macrophage polarization markers (decreased iNOS and increased ARG1 protein)​. Iba1 immunofluorescence further revealed that 3-BrPA treatment was associated with reduced aggregation and a less activated morphology of microglia. When interpreting these results, it is important to acknowledge two limitations. First, our characterization of microglial polarization remains relatively limited, as more definitive immune profiling approaches—such as flow cytometry for surface markers (e.g., CD86/CD206) or single-cell RNA sequencing—were not performed. Second, and central to the framing of our conclusion, the reliance on pharmacological inhibition precludes definitive causal attribution.​ Collectively, these data link the pharmacological perturbation of the *Hk2*-glycolysis axis to attenuated neuroinflammation and tissue damage, supporting the association of *Hk2* with a glycolytic-inflammatory axis in HIBD (6, 47).

The timing and dosage of intervention are critical considerations for metabolic modulation. We found that a single administration of 3-BrPA at 1 hour post-HIBD was associated with a dose-dependent reduction in acute cerebral infarction volume, with the 1 mg/kg dose showing the most pronounced effect. Interestingly, the effect was diminished at the 2 mg/kg dose, presenting a non-monotonic dose-response relationship. This suggests a potential narrow therapeutic window for glycolytic inhibition *in vivo*, where excessive inhibition may lead to reduced efficacy or off-target effects. The optimal dose we observed (1 mg/kg) is lower than the dose (5 mg/kg) used in an adult focal stroke model (18). This discrepancy may relate to fundamental differences between neonatal global HIBD and adult focal ischemia models in terms of basal metabolism, the developing blood-brain barrier, the immature immune system, and the unique metabolic vulnerabilities of the developing brain. Our finding highlights the importance of optimizing therapeutic strategies within the specific context of the developing brain (53, 54). This early metabolic intervention was associated with lasting benefits as assessed by *in vivo* metrics. A single dose of 3-BrPA was associated not only with attenuated acute injury but also with reduced long-term brain tissue atrophy and improved motor coordination and exploratory behavior at 30 days post-injury. We explicitly state that chronic histological validation (e.g., assessment of neuronal survival, synaptic density, or white matter integrity) was not performed. Therefore, conclusions regarding the cellular correlates of persistent neuroprotection remain primarily supported by these *in vivo* imaging (MRI) and behavioral outcomes.​ Nonetheless, the observed preservation of brain structure and function suggests that modulating the acute metabolic-immune imbalance may have a sustained positive impact, warranting further investigation into its effects on neural circuit development and long-term neurodevelopmental outcomes.

This study has several limitations. First, we relied on 3-BrPA as a pharmacological tool. Although it is often discussed as a relatively selective agent in this context, it is not entirely specific and may affect other glycolytic enzymes or possess alkylating effects (55, 56). Future studies utilizing conditional genetic tools are needed to more precisely define the role of *Hk2* in specific cell types (41). Second, the interpretation of CIBERSORT data in brain tissue requires caution, as it reflects relative RNA abundances rather than absolute cell counts (57). Third, as a preliminary study, we used mixed-sex cohorts. Future work should investigate potential sex-dependent differences (58, 59).

## 5 Conclusion

In summary, our integrated analysis highlights *Hk2* upregulation in microglia as a feature of HIBD. The use of 3-BrPA is associated with modulation of this pathway, concomitant reductions in glycolytic flux and neuroinflammation, and improved long-term functional and imaging outcomes. These observations link acute metabolic reprogramming to neuroinflammatory pathology in HIBD and highlight the therapeutic potential of targeting the *Hk2*-associated metabolic axis.

## Data Availability

The datasets presented in this study can be found in online repositories. The names of the repository/repositories and accession number(s) can be found in the article/**Supplementary Material**.

## Supplementary material

Supplementary Figure 1 GSEA based on core gene expression stratification. **(A, B)** Hallmark pathways enriched in high *vs*. low *Hk2* expression groups. **(C, D)** Hallmark pathways enriched in high *vs*. low *Egfr* expression groups. **(E, F)** Hallmark pathways enriched in high *vs*. low *Vegfa* expression groups.
