# Supplementary figures and images for: Hexokinase 2 upregulation is associated with glycolytic reprogramming and neuroinflammation in hypoxic-ischemic brain damage: a therapeutic target for early intervention
Source: Front Immunol. 2026 Jun 12;17:1837728. doi: 10.3389/fimmu.2026.1837728 (PMC13303037; doi:10.3389/fimmu.2026.1837728)

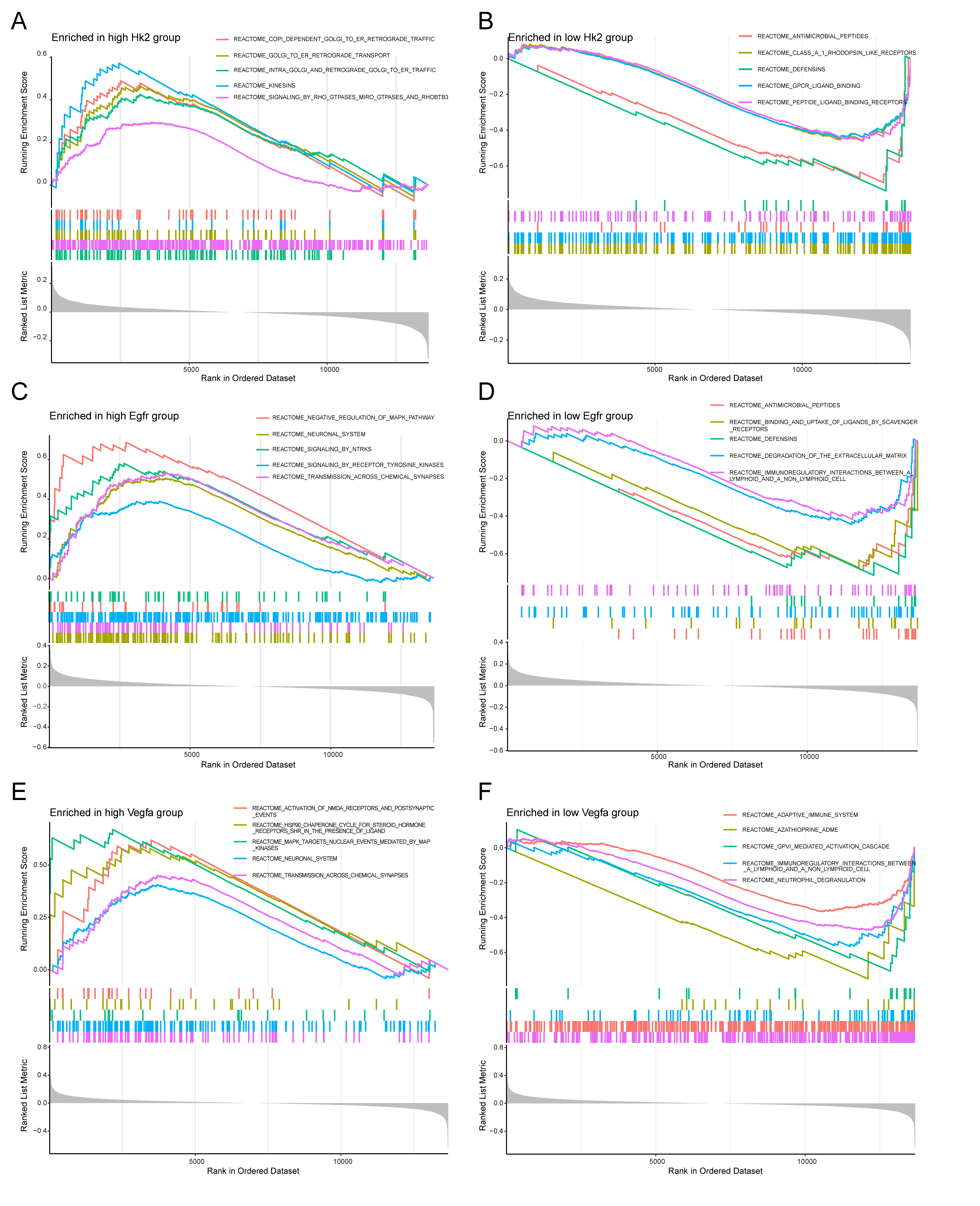

Supplement: Supplementary Figure 1 — GSEA based on core gene expression stratification. (A, B) Hallmark pathways enriched in high vs. low Hk2 expression groups. (C, D) Hallmark pathways enriched in high vs. low Egfr expression groups. (E, F) Hallmark pathways enriched in high vs. low Vegfa expression groups. [file Image1.tif]
