# Supplementary material for: Hexokinase 2 upregulation is associated with glycolytic reprogramming and neuroinflammation in hypoxic-ischemic brain damage: a therapeutic target for early intervention
Source: Front Immunol. 2026 Jun 12;17:1837728. doi: 10.3389/fimmu.2026.1837728 (PMC13303037; doi:10.3389/fimmu.2026.1837728)
Supplement: Supplementary file 5 [file Table1.docx]

| Data set | Platform | Organism | Type | Samples (Control/HIBD) | Tissue |
| --- | --- | --- | --- | --- | --- |
| GSE144456 | GPL10333 | Mus musculus | mRNA | P5, P10  3h:3/3  8h:3/3  12h:3/3  24h:3/3 | Forebrain |
| GSE23317 | GPL6885 | Mus musculus | mRNA | P8  3h:4/3  8h:3/4  24h:4/4 | Cerebral cortex |
